# Bronchoscopic removal of a leech from the trachea by cryotherapy

**DOI:** 10.1111/crj.13522

**Published:** 2022-07-08

**Authors:** Dengyuan Li, Danxiong Sun

**Affiliations:** ^1^ Department of Respiratory and Critical Care Medicine First People's Hospital of Yunnan Province Kunming China; ^2^ Faculty of Life science and technology Kunming University of Science and Technology Kunming China

**Keywords:** bronchoscope, cryotherapy, leech infestation, trachea

## Abstract

This report presents a 74‐year‐old man with haemoptysis, cough and pharyngeal discomfort. Bronchoscopy revealed a brown worm‐like moving foreign body attaching to the upper trachea approximately 2 cm below the glottis. This worm was identified as a leech, measured about 4 cm and was mobile. It was removed safely in one piece via cryotherapy by flexible bronchoscope. Intrabronchial cryotherapy by bronchoscope might be the best way of extraction of leeches.

## Graphical abstract

A 74‐year‐old man presented to our hospital with complaints of haemoptysis, cough and pharyngeal discomfort for 45 days. Bronchoscopy revealed a leech attaching to the upper trachea approximately 2 cm below the glottis. The leech was removed safely in one piece via cryotherapy by flexible bronchoscope under local anaesthesia.

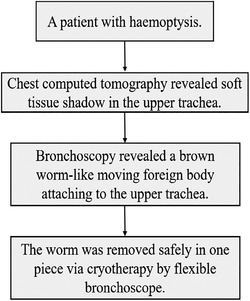

The tracheal leech infestation is a relatively rare cause of haemoptysis. Traditionally, leeches were removed by using forceps.^1^ Because leeches attach to the mucosa by strong suction, rupture of the worm is a risk of the traditional way.

Here, we reported a rare case of haemoptysis whose cause has proven an infestation of the trachea by a leech, which was removed safely in one piece via cryotherapy by flexible bronchoscope. A 74‐year‐old man presented to our hospital with complaints of haemoptysis, cough and pharyngeal discomfort for 45 days. He had no dyspnoea, fever, purulent sputum, dysphagia, epistaxis or melena. The patients came from rural area and had a history of drinking unpurified water from streams.

On physical examination, there were no abnormal findings, and vital signs were normal. He had good general conditions. No cyanosis of lip. No subcutaneous haemorrhage. Both lungs were clear. Routine blood test, biochemical parameters, coagulation function, stool test and urine analysis were normal. Chest computed tomography revealed some soft tissue shadow in the upper trachea (Figure 1A).

**FIGURE 1:**
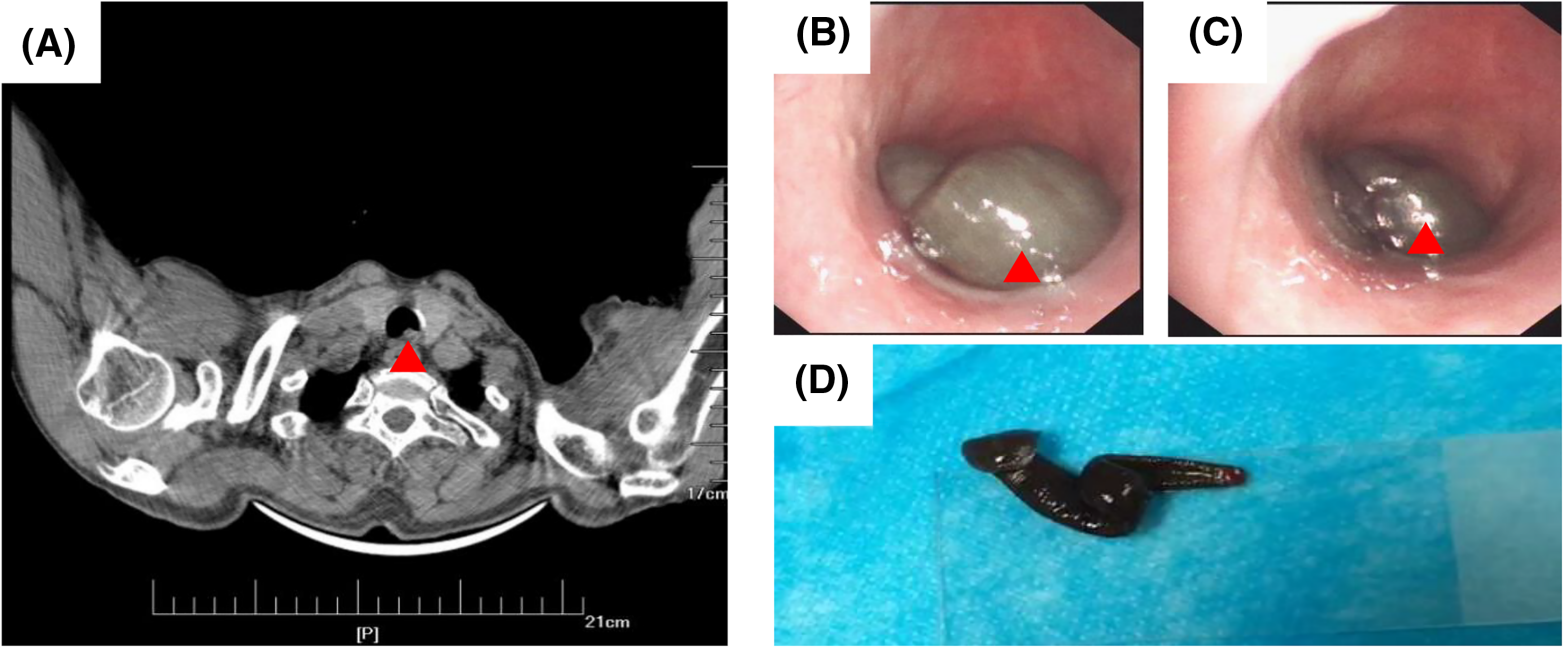
(A) Computed Tomography (CT) showing soft tissue shadow in the upper trachea. (B,C) The leech in the upper trachea. (D) The leech was removed from the patient.

Bronchoscopy was performed following nasal packing under local anaesthesia and oxygen. After 1% lidocaine was sprayed into the larynx, glottis and upper trachea, flexible bronchoscope was inserted into the trachea. It revealed a brown worm‐like moving foreign body attaching to the upper trachea approximately 2 cm below the glottis (Figure 1B,C). The flexible cryoprobe was passed through the working channel of the bronchoscope. The cryoprobe tip was directly applied to the anterior sucker. The cryoprobe began with a foot pedal. About 5 s later, an ice ball appeared on the tip of the probe, which meant that the probe clung tightly to the sucker and the sucker lost adhesion and then removed the probe and bronchoscope quickly. This worm was identified as a leech, measured about 4 cm and was mobile (Figure 1D). Repeat bronchoscopy revealed no bleeding and blood clots in the trachea.

The symptoms were improved obviously after removal of the leech. One week after discharge, by telephone follow‐up, the patient did not complain of discomfort.

Leeches are blood‐sucking parasites that are generally found in puddles of water and streams. They have two suckers. The posterior sucker is mainly used for leverage, whereas the anterior one, which consists of the jaw and teeth, is where the feeding takes place. Leeches can reach the lower airway when water is ingested directly from infected lakes or rivers.

The extraction of the leech attached to the respiratory tract is not always easy, not only because of its slippery body surface that can easily be ruptured but also due to its important link power. A case report described a 15‐year‐old female from Iran whose leech infestation was located in the right lower lobe anteromedial bronchus.^2^ When the doctors tried to remove the worm by forceps, it ruptured, whereas the leech's sucker remained attached to the bronchial mucosa. The remaining worm particle was removed by using a Dormia basket. In addition, a case was a 40‐year‐old woman from China with tracheal leech infestation.^3^ The doctors performed bronchoscopy under lidocaine topical anaesthesia, and intended to remove it by using forceps, but failed to extract it. Eventually, the leech was removed by rigid bronchoscopy under general anaesthesia.

Foreign bodies retrieval by cryoprobe is feasible for many organic and inorganic aspirated objects.^4^ As early as 1989, Roden and Homasson ^5^ first reported the successful removal of foreign bodies via intrabronchial cryotherapy by rigid bronchoscopy. The ability to remove a foreign body by cryoextraction depends on the cryo‐adhesive properties of the retrieved object. As a live foreign body abundant with water, the leech can be easily removed by cryoprobe in theory. In our case, the leech was removed safely in one piece by using intrabronchial cryotherapy with the requirement of local anaesthesia. Cryotherapy causes the leech's sucker to lose adhesion, making it easier to extract.

In conclusion, the physician should be aware of the possibility of the tracheobronchial leeches if the patients have a history of drinking unpurified water in fields and have the following symptoms, such as haemoptysis, cough and throat discomfort. Intrabronchial cryotherapy by bronchoscope might be the best way of extraction of leeches.

## CONFLICT OF INTEREST

The authors have no conflicts of interest to declare.

## ETHICS STATEMENT

The study was conducted in accordance with the principles of the Declaration of Helsinki and Good Clinical Practice guidelines. The study was approved by the Ethics Committee of First hospital of Yunnan Province. Written informed consent was obtained from individual. Written informed consent has been obtained from the relative of the participant, who is approved the publication of the manuscript with anonymity.

## AUTHOR CONTRIBUTIONS

All authors contributed to the acquisition of data, writing and revision of this manuscript.

## Data Availability

All supporting data are included in this article.
